# Association Between Handgrip Strength and Mortality of Patients With Coronary Artery Disease: A Meta‐Analysis

**DOI:** 10.1002/clc.24322

**Published:** 2024-07-25

**Authors:** Meiling Xiao, Yu Lu, Hongqiu Li, Zhonghai Zhao

**Affiliations:** ^1^ Department of Rehabilitation Central Hospital Affiliated to Shenyang Medical College Shenyang China; ^2^ Department of Rehabilitation Medicine The People's Hospital of Liaoning Province Shenyang China

**Keywords:** coronary artery disease, handgrip strength, meta‐analysis, mortality, risk factor

## Abstract

**Background:**

Muscular strength has been linked to increased risk of cardiovascular disease in the community population. The aim of this systematic review and meta‐analysis is to evaluate the association between weak handgrip strength (HGS) and mortality risk in patients with coronary artery disease (CAD).

**Methods:**

To carry out the meta‐analysis, an extensive search was conducted on databases such as PubMed, Embase, Web of Science, Cochrane Library, Wanfang, and CNKI to identify observational studies with longitudinal follow‐up. Random‐effects models were used to combine the findings, taking into account the potential influence of heterogeneity.

**Results:**

Eight observational studies involving 10 543 patients with CAD were included. During a mean follow‐up duration of 20.4 months, 1327 (12.6%) patients died. Pooled results showed that weak HGS at baseline was associated with an increased risk of all‐cause mortality during follow‐up (risk ratio [RR]: 1.95, 95% confidence interval: 1.50 to 2.55, *p* < 0.001; *I* ^2^ = 62%). Subgroup analysis suggested a stronger association between weak HGS and increased mortality in older patients with CAD as compared to that of overall adult patients with CAD (RR: 3.01 vs. 1.60, *p* for subgroup difference = 0.004). Subgroup analyses according to study location, design, subtype of CAD, follow‐up duration, analytical model, and study quality scores showed similar results (*p* for subgroup difference all > 0.05).

**Conclusions:**

Weak HGS at baseline is associated with an increased risk of mortality in patients with CAD, particularly in older patients with CAD.

## Graphical abstract

A meta‐analysis showed that weak handgrip strength at baseline is associated with an increased risk of mortality in patients with coronary artery disease, particularly in older patients. In view of the convenience, noninvasive nature, and repeatability of the methodology, measurement of handgrip strength should be incorporated into the evaluation and management of patients with coronary artery disease.

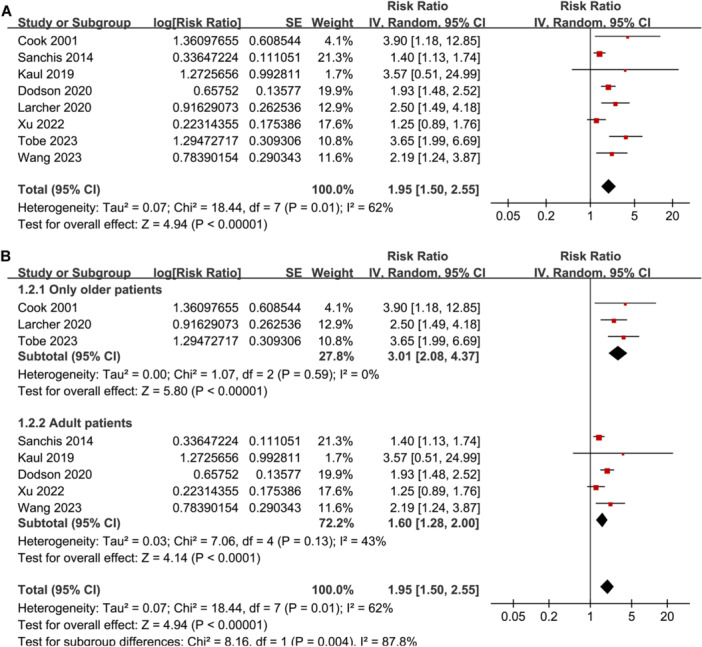

## 1 Background

Despite the diagnostic and therapeutic advances in recent decades, coronary artery disease (CAD) remains a leading cause of morbidity and mortality globally [1]. Pathophysiologically, CAD is characterized by atherosclerotic stenosis or occlusion of the coronary artery, leading to ischemia and necrosis of the myocardium [2, 3]. According to the clinical presentation, CAD can be classified as acute coronary syndrome (ACS) [4] and stable CAD [5]. Although effective treatments such as coronary revascularization have significantly improved the prognosis of patients with CAD, the incidences of heart failure and other adverse cardiovascular events remain high in these patients, leading to an overall increased mortality [6]. Accordingly, it is important to determine clinical factors that are associated with the poor prognosis of patients with CAD.

Chronic inflammation has been recognized as a key mechanism during the pathogenesis and deterioration of CAD [7]. On the other hand, persistent systemic inflammation also leads to reduction of peripheral muscular strength and mass, which is known as sarcopenia [8]. Clinically, handgrip strength (HGS) is a validated indicator of muscle strength in the adult population that can be conveniently measured using a muscle strength dynamometer [9, 10]. Accumulating evidence suggests that a reduction in HGS may confer higher risk of cardiovascular diseases [11, 12, 13, 14]. However, it has not yet been fully determined if weak HGS in patients with CAD could predict a poor prognosis and previous studies that have evaluated the association have reported inconsistent results. Some of the previous studies showed that weak HGS may be associated with an increased risk of mortality in patients with CAD [15, 16, 17, 18, 19, 20], while others did not show similar results [21, 22]. Therefore, in this study, we performed a systematic review and meta‐analysis to evaluate the association between weak HGS and mortality risk in patients with CAD.

## 2 Materials and Methods

The research followed the Meta‐analyses Of Observational Studies in Epidemiology (MOOSE) guideline [23] during the phases of planning, execution, and documentation.

### 2.1 Inclusion and Exclusion Criteria of Studies

The development of inclusion criteria adhered to the PICOS recommendations and aligned with the objective of the meta‐analysis.

P (patients): Patients with confirmed diagnosis of CAD, regardless of the subtype.

I (exposure): Patients with weak HGS at baseline, which was measured using a muscle strength dynamometer. The cutoff for defining weak HGS was consistent with the criteria used among the included original studies.

C (control): Patients with normal HGS at baseline.

O (outcomes): Incidence of all‐cause mortality of patients with CAD, as compared between patients with and without weak HGS at baseline.

S (study design): Observational studies with longitudinal follow‐up, such as cohort studies, post‐hoc analysis of clinical studies, and nested case–control studies.

Literature reviews, editorials, meta‐analyses, and studies that did not involve patients with CAD, did not assess HGS as an exposure variable, or did not report the outcome of mortality during follow‐up were excluded from the meta‐analysis. In instances where there was a duplication of patient populations, the study with the most extensive sample size was incorporated into the meta‐analysis.

### 2.2 Search of Databases

Studies relevant to the objective of the meta‐analysis were identified by search of electronic databases, namely, PubMed, Embase, Web of Science, Cochrane Library, Wanfang, and China National Knowledge Infrastructure (CNKI), encompassing the period from inception to October 8, 2023. Cochrane Library was searched because the post‐hoc analyses of clinical trials that fit the aim of the meta‐analysis could also be included. The search strategy used relevant terms pertaining to the subject matter of our investigation, aiming to identify studies published within this timeframe, which included (1) “handgrip” OR “hand strength” OR “muscle strength dynamometer” OR “grip strength”; (2) “coronary artery disease” OR “coronary heart disease” OR “ischemic heart disease” OR “CAD” OR “CHD” OR “percutaneous coronary intervention” OR “PCI” OR “acute myocardial infarction” OR “AMI” OR “myocardial infarction” OR “MI” OR “STEMI” OR “ST‐Elevation” OR “non‐ST segment elevation” OR “NSTEMI” OR “ACS” OR “acute coronary syndrome”; and (3) “mortality” OR “died” OR “death” OR “prognosis” OR “follow” OR “follow‐up” OR “prospective” OR “retrospective” OR “longitudinal” OR “cohort” OR “incidence” OR “risk” OR “recurrence”. Only full‐length articles published in peer‐reviewed journals in English or Chinese were included. Gray literature (conference abstracts or unpublished data) was not considered because it was generally not peer‐reviewed. Additionally, during our manual screening process, we thoroughly examined the references cited in relevant original and review articles to identify any potentially relevant studies.

### 2.3 Data Extraction and Quality Evaluation

Two authors conducted literature searches, collected data, and assessed the quality of the studies separately. In instances where inconsistencies arose, the authors engaged in discussions to reach a consensus. The analysis of the studies involved gathering data pertaining to study details, design attributes, sample size, patient demographics, subtype of CAD, cutoff for evaluating HGS, duration of follow‐up, number of patients who died during follow‐up in each study, and potential confounding factors adjusted when the association between HGS and mortality of patients with CAD was analyzed. The quality of the study was evaluated using the Newcastle–Ottawa Scale (NOS) [24]. This scale assesses the quality of cohort studies based on three dimensions: selection of study groups, comparability of these groups, and ascertainment of the outcome of interest. The NOS varied between one and nine stars, with a higher star indicating better study quality.

### 2.4 Statistics

Risk ratios (RRs) and their corresponding 95% confidence intervals (CIs) were utilized as the variables to assess the relationship between weak HGS and mortality risk of patients with CAD. For studies that reported the hazard ratio (HR), HR was directly considered as RR, while for studies that reported the odds ratio (OR), data were converted into RR for the meta‐analysis as previously reported (RR = OR/([1 − pRef] + [pRef × OR]), where pRef is the prevalence of the outcome in the reference group (normal HGS group) [25]. In order to stabilize and standardize the variance, a logarithmic transformation was implemented on the OR and its corresponding standard error in each study [26]. The Cochrane *Q* test and the *I* ^2^ statistic [27] were utilized to assess between‐study heterogeneity. A value of *I* ^2^ exceeding 50% signifies the existence of substantial heterogeneity among the studies. The random‐effects model was used for synthesizing the results, as it is acknowledged for its ability to accommodate potential heterogeneity [28]. Sensitivity analysis excluding one data set at a time was performed to evaluate the robustness of the finding. Additionally, predefined subgroup analysis was conducted to explore whether the results were significantly affected by the age group of the patients (older or overall adult patients), study country, design, subtype of CAD (ACS or stable CAD), follow‐up duration, analytic models (univariate or multivariate), and quality scores of the study. Publication bias was estimated using a funnel plot, which involved visual assessments of symmetry, as well as Egger's regression asymmetry test [29]. The statistical analyses were conducted using RevMan (Version 5.1; Cochrane Collaboration, Oxford, UK) and Stata software (Version 12.0; Stata Corporation, College Station, TX).

## 3 Results

### 3.1 Database Search and Study Retrieval

**Figure 1:**
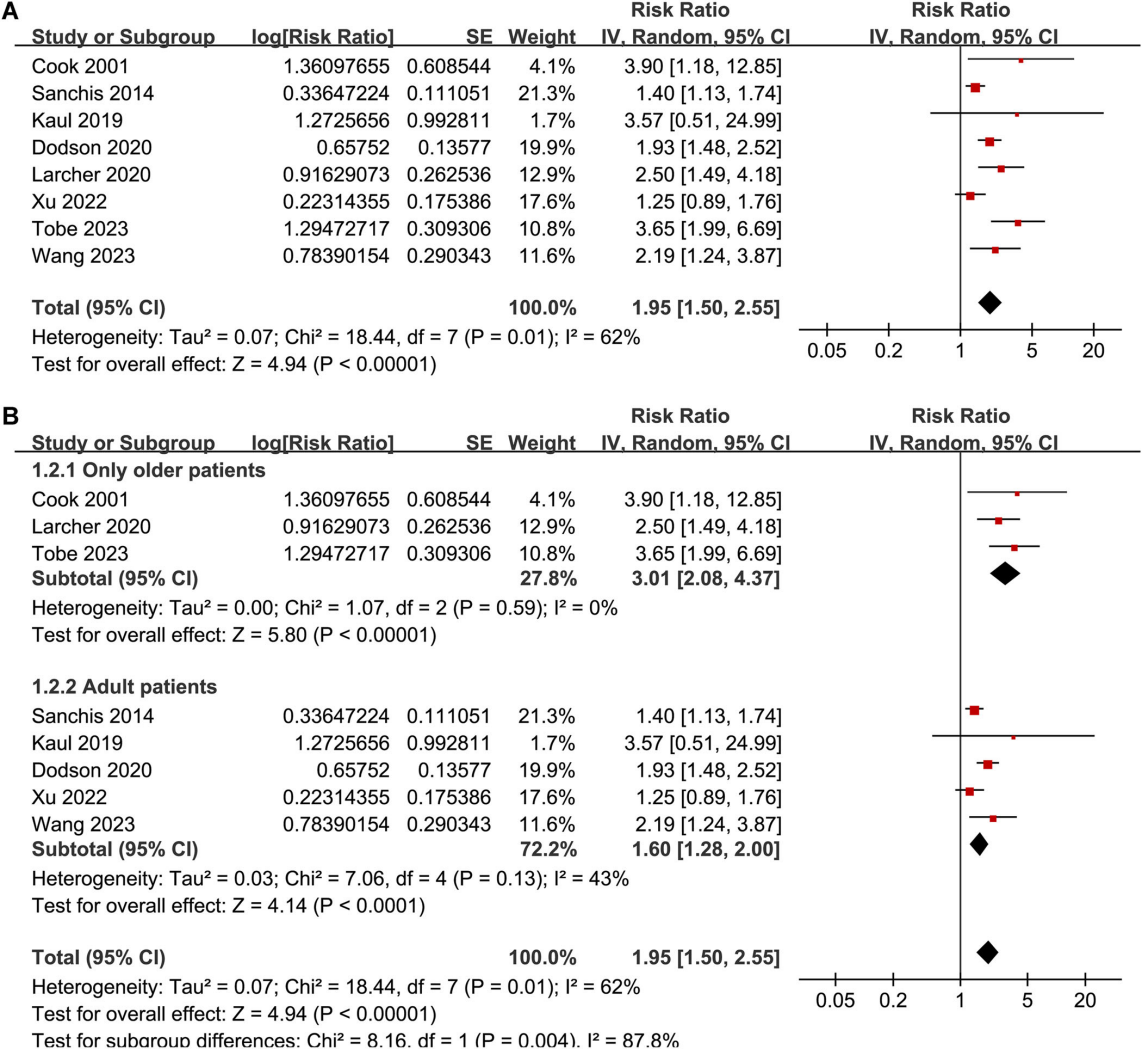
Forest plots for the meta‐analysis regarding the association between weak HGS and mortality risk of patients with CAD. (A) Overall meta‐analysis and (B) subgroup analysis according to age of the patients.

Figure 1 illustrates the procedure used to conduct the literature search and study retrieval. Initially, a total of 685 records were acquired from the designated database, and subsequently, 147 duplicate entries were eliminated. Upon scrutinizing the titles and abstracts, an additional 517 studies were excluded due to their incompatibility with the objectives of the meta‐analysis. Following comprehensive evaluations of the full texts of 21 studies, 13 were excluded based on the rationales outlined in Figure S1. Consequently, eight studies [15, 16, 17, 18, 19, 20, 21, 22] were deemed suitable for the subsequent meta‐analysis.

### 3.2 Study Characteristics

Overall, eight studies, including five prospective cohort studies [15, 16, 17, 18, 19], two retrospective cohort studies [20, 22], and one post‐hoc analysis of clinical trial [21], were included in the meta‐analysis. The characteristic of the studies are summarized in Table 1. These studies were conducted in the United States, Spain, Canada, Austria, China, and Japan, and were published within the timeframe of 2001 to 2023. Three studies included patients with acute myocardial infarction or ACS only [16, 17, 21], one study included patients with stable CAD only [18], while another four studies included both ACS and stable CAD patients [15, 19, 20, 22]. Four of the studies included patients with CAD aged 65 years or older [16, 17, 21, 22], while the other four studies included overall adult patients with CAD [15, 18, 19, 20]. Cutoffs for defining weak HGS also differed among the included studies. Six studies had a cutoff of 26–32 kg for men and 18–25 kg for women [15, 17, 19, 20, 21, 22], while the other two studies compared HGS between quartiles [16, 18]. The follow‐up duration was 3–110 months (mean: 20.4 months), and 1327 (12.6%) patients died during follow‐up. A univariate analysis was carried out in two studies [15, 17] when the association between HGS and mortality of patients with CAD was analyzed, while for the other six studies [16, 18, 19, 20, 21, 22], multivariate analysis was carried out. Potential confounding factors such as age, sex, cardiovascular risk factors, comorbidities, and treatments were adjusted in the multivariate analysis. The NOS of these studies ranged from 6 to 9, indicating their high quality (Table S1).

**Table 1:** Characteristics of the included studies.

Study	Location	Study design	Patient status	No. of patients	Mean age (years)	Men (%)	ACS (%)	HGS cutoff	Follow‐up duration (months)	No. of patients died	Variables adjusted
Cook [15]	USA	PC	CAD patients after CABG	200	NR	73	51	Men < 30 kg, women < 25 kg	3	11	None
Sanchis [16]	Spain	PC	ACS patients aged over 65	342	77	57	100	Sex‐based quartiles	30	74	Age, sex, smoking, comorbidities, hemodynamic and ECG data, troponin elevation, HGB, eGFR, LVEF, and invasive management data
Kaul [21]	Canada	Post‐hoc analysis	ACS patients aged over 65	4996	72.9	53.9	100	Men < 32 kg, women < 21 kg	17.3	619	Age, sex, weight, region, ACS classification, Killip class, BP, HR, time to treatment, comorbidities, prior PCI, prior CABG, HGB, SCr, angiography before randomization, treatment, and concomitant medications
Dodson [17]	USA	PC	AMI patients aged over 75	3006	81.5	44.4	100	Men < 28.5 kg, women < 18.5 kg	6	266	None
Larcher [18]	Austria	PC	Patients with angiographically proven stable CAD	691	65.4	71.1	0	Quartiles	110	216	Age, sex, BMI, T2DM, HTN, history of smoking, TC, LDL‐C, and HDL‐C
Xu [22]	China	RC	CAD patients aged over 65	677	75	56.8	27.4	Men < 28 kg, women < 18 kg	12	47	Age, sex, T2DM, malnutrition, ACS, and gait speed
Tobe [19]	Japan	PC	CAD patients undergoing PCI	469	72.3	81.8	27.1	Men < 28 kg, women < 18 kg	25.6	41	Age, sex, BMI, comorbidities, eGFR, history of smoking, HGB, serum albumin, LVEF, previous MI, PCI and CABG, and ACS
Wang [20]	China	RC	Patients with angiographically proven stable CAD aged over 65	162	73.6	57.4	55	Men < 26 kg, women < 18 kg	24	53	Age, sex, LVEF, TC, serum albumin, malnutrition, and ACS

Abbreviations: ACS, acute coronary syndrome; AMI, acute myocardial infarction; BMI, body mass index; BP, blood pressure; CABG, coronary artery bypass graft; CAD, coronary artery disease; ECG, electrocardiograph; eGFR, estimated glomerular filtrating rate; HDL‐C, high‐density lipoprotein cholesterol; HGB, hemoglobin; HGS, handgrip strength; HR, heart rate; HTN, hypertension; LDL‐C, low‐density lipoprotein cholesterol; LVEF, left ventricular ejection fraction; MI, myocardial infarction; NR, not reported; PC, prospective cohort; PCI, percutaneous coronary intervention; RC, retrospective cohort; SCr, serum creatinine; T2DM, type 2 diabetes mellitus; TC, total cholesterol.

### 3.3 Results of Meta‐Analysis

**Figure 2:**
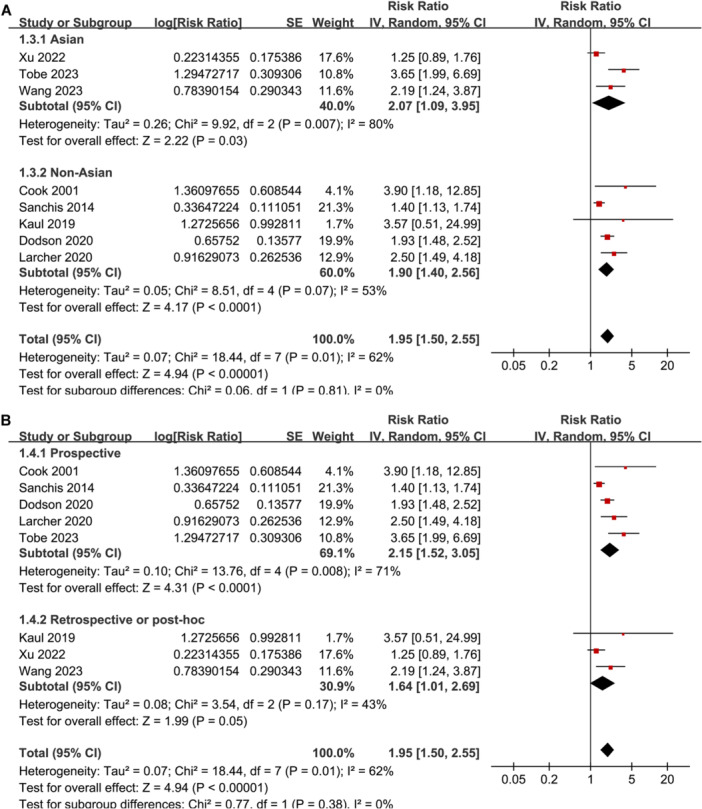
Forest plots for the subgroup analyses regarding the association between weak HGS and mortality risk of patients with CAD. (A) Subgroup analysis according to study country and (B) subgroup analysis according to study design.

**Figure 3:**
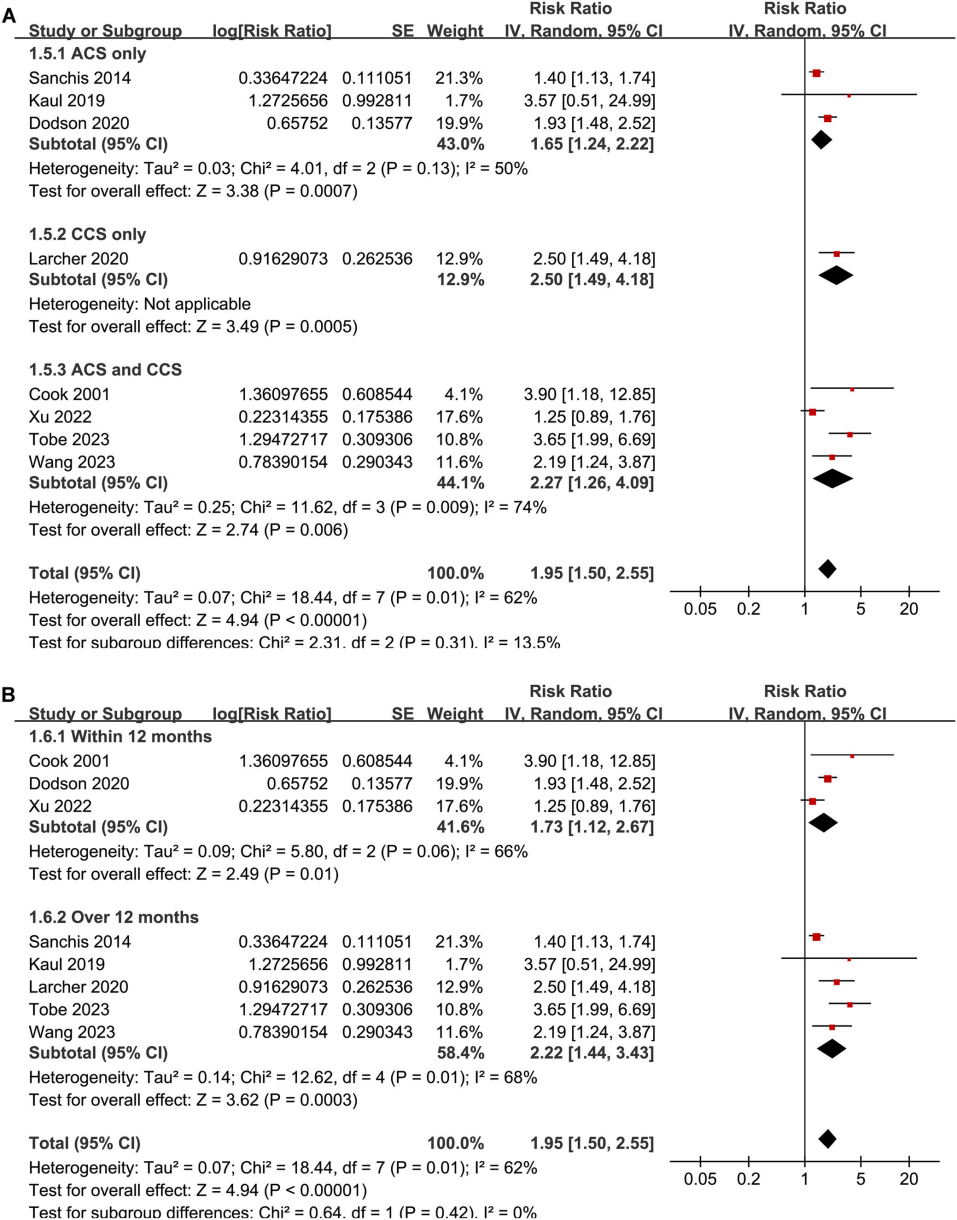
Forest plots for the subgroup analyses regarding the association between weak HGS and mortality risk of patients with CAD. (A) Subgroup analysis according to subtype of CAD and (B) subgroup analysis according to follow‐up duration.

**Figure 4:**
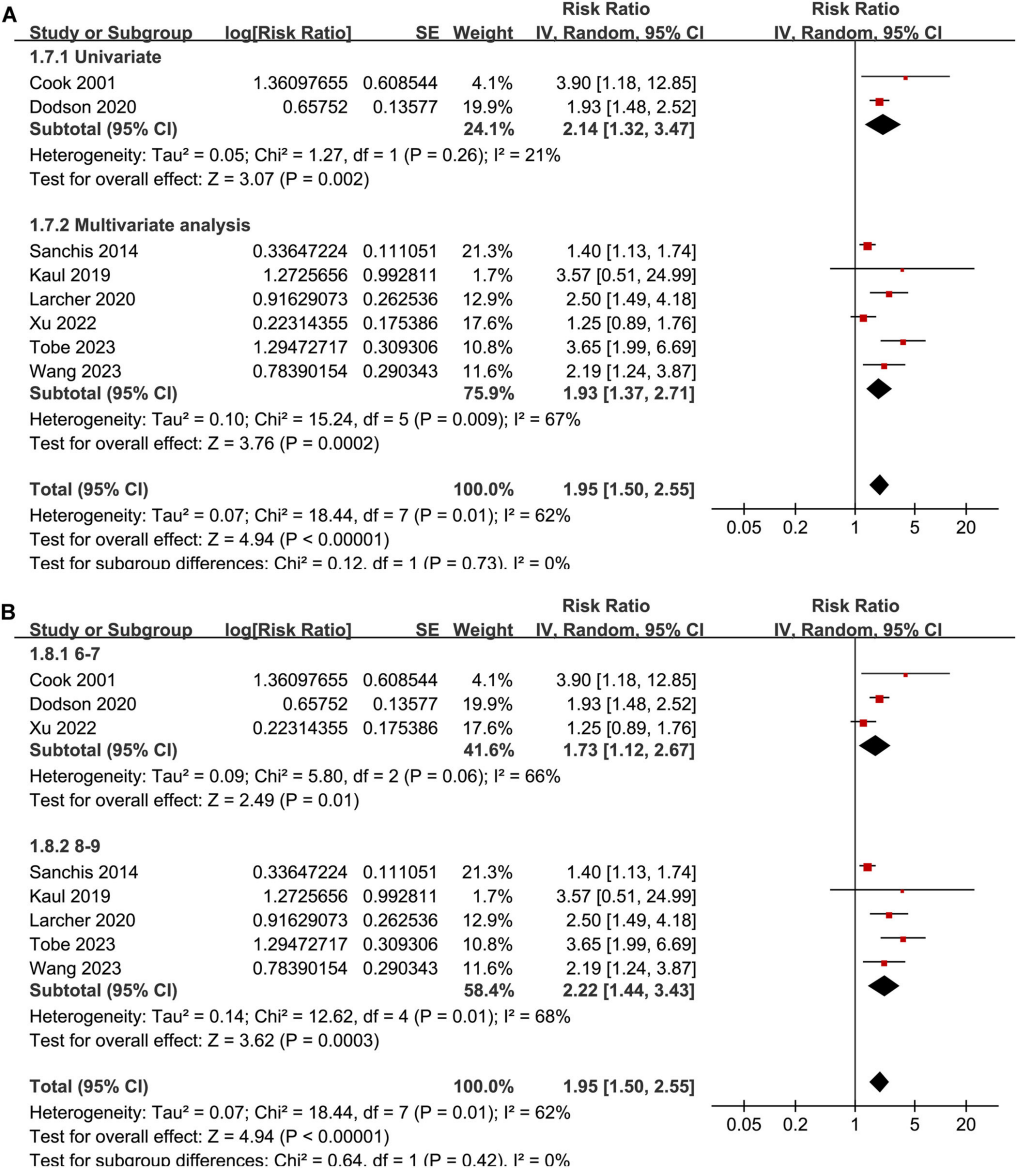
Forest plots for the subgroup analyses regarding the association between weak HGS and mortality risk of patients with CAD. (A) Subgroup analysis according to analytic models and (B) subgroup analysis according to study quality scores.

Pooled results showed that weak HGS at baseline was associated with an increased risk of all‐cause mortality during follow‐up (RR: 1.95, 95% CI: 1.50 to 2.55, *p* < 0.001; *I* ^2^ = 62%; Figure 1A). Sensitivity analyses by excluding one study at a time showed consistent results (RR: 1.63 to 1.93, *p* all < 0.05). Subgroup analysis suggested a stronger association between weak HGS and increased mortality in older patients with CAD (RR: 3.01, 95% CI: 2.08 to 4.37, *p* < 0.001; *I* ^2^ = 0%) as compared to that of overall adult patients with CAD (RR: 1.60, 95% CI: 1.28 to 2.00, *p* < 0.001; *I* ^2^ = 43%; *p* for subgroup difference = 0.004; Figure 1B). Subgroup analyses according to study location (*p* for subgroup difference = 0.81; Figure 2A), study design (*p* for subgroup difference = 0.38; Figure 2B), subtype of CAD (*p* for subgroup difference = 0.31; Figure 3A), follow‐up duration (*p* for subgroup difference = 0.42; Figure 3B), analytic model (*p* for subgroup difference = 0.73; Figure 4A), and study quality scores (*p* for subgroup difference = 0.42; Figure 4B) showed similar results.

### 3.4 Publication Bias

The funnel plots depicting the meta‐analyses of the association between weak HGS and the mortality risk of patients with CAD are presented in Figure S2. Upon visual inspection, the plots show symmetrical patterns, indicating minimal presence of publication bias. Furthermore, the application of Egger's regression tests yielded a *p* value of 0.18, further supporting the notion of a low probability of publication bias.

## 4 Discussion

In this systematic review and meta‐analysis, we integrated the evidence from eight longitudinal observational studies, and the results showed that a decreased HGS at baseline was associated with an increased risk of all‐cause mortality in patients with CAD. Further subgroup analysis according to the age group of the patients showed that the association may be stronger in older patients with CAD as compared to that in overall adult patients with CAD. Finally, subgroup analyses according to study location, design, subtype of CAD, follow‐up duration, analytic model, and study quality scores showed similar results. Taken together, results of the meta‐analysis indicate that reduced muscular strength as evidenced by lower HGS at baseline may be a risk factor of all‐cause mortality in patients with CAD, particularly for older patients.

To the best of our knowledge, this study may be the first meta‐analysis examining the correlation between HGS and the risk of all‐cause mortality in patients diagnosed with CAD. It is important to acknowledge several methodological strengths before interpreting the findings. First, a comprehensive search was conducted across six widely utilized electronic databases to identify relevant studies, resulting in the retrieval of current literature investigating the potential prognostic significance of HGS in CAD patients. Second, all included studies were observational in nature and featured longitudinal follow‐up, thereby providing a longitudinal relationship between a lower HGS and increased all‐cause mortality in these patients. Third, the sensitivity analyses conducted by systematically excluding individual studies consistently yielded results that reinforce the reliability and validity of the findings. Additionally, multiple subgroup analyses were conducted to assess the relationship between HGS and mortality in patients with CAD. The consistent outcomes obtained from these subgroup analyses further validate that the association remains unaffected by various study characteristics.

The potential mechanisms underlying the association of weak HGS and increased mortality risk in patients with CAD may be multifactorial. First, in a previous cross‐sectional study of patients with ST‐segment elevation myocardial infarction (STEMI), a reduced HGS at admission was independently correlated with the severity of coronary lesions as evidenced by high Gensini scores [30]. In addition, STEMI patients with weak HGS had a higher incidence of no‐reflow [30], suggesting that a reduced HGS may be a marker of increased atherosclerotic burden in these patients. In addition, weak HGS has been incorporated as important components of sarcopenia [31] and frailty [32], two established risk factors of poor prognosis of patients with CAD, which may also partly explain the relationship between a low HGS and increased risk of patients with CAD. Moreover, a low HGS has been related to endothelial dysfunction and activated systematic inflammatory response, two key elements involved in the deterioration of atherosclerosis [33, 34]. Finally, a reduced muscular strength is associated with reduced functional capacity (peak oxygen consumption), deteriorating symptoms of dyspnea, and increased risks of adverse events such as falls, osteoporosis, and fracture, which all increase the risk of mortality in patients with cardiovascular disease, particularly for the older patients [35]. This may also be the reason for the results of the subgroup analysis, showing a stronger relationship between low HGS and mortality risk in older patients with CAD. Further research is necessary to elucidate the underlying mechanisms linking a low HGS and mortality in patients with CAD. However, the results of the meta‐analysis provide support for the inclusion of HGS assessment in risk stratification for the management of CAD patients. From a clinical standpoint, measuring HGS in CAD patients is a convenient, cost‐effective, and replicable method, further bolstering its potential as a prognostic tool. Additionally, it is crucial to investigate whether interventions aimed at improving HGS can effectively reduce mortality in this population. Studies are warranted in the future for further investigation.

This meta‐analysis also has limitations. First, the numbers of included studies and the sample size were small, and the results should be validated in large‐scale prospective studies. Moreover, significantly heterogeneity was observed among the included studies. Although we performed a series of sensitivity and subgroup analyses, the source of the heterogeneity remains to be determined. In addition, the limited number of included studies did not allow us to perform meta‐regression analysis to further evaluate the potential influencing factors. It should be noticed that the cutoffs for defining the weak HGS and the potential confounding factors adjusted were variable among the included studies, which may potentially lead to heterogeneity. Future studies are needed to determine the optimal cutoff of HGS for predicting the mortality risk in patients with CAD. Besides, the sole focus on weak HGS overlooks the importance of examining the association of “normal” or strong HGS with all‐cause mortality, especially in older patients. We were unable to determine if CAD patients with strong HGS had better prognosis as compared to those with normal HGS, because none of the included studies had a group of patients with strong HGS. However, two previous clinical studies showed that exercise training that improved HGS in older patients with CAD was associated with improved exercise capacity as indicated by the findings of peak oxygen consumption in cardiopulmonary exercise testing [36] and the 6‐min walk test [37]. Since these two parameters are closely related to the mortality risk of patients with CAD [38, 39], these findings may suggest that enhancing HGS could improve the prognosis of patients with CAD. Studies are warranted for confirmation of the hypotheses. In addition, this meta‐analysis was based on study‐level data rather than individual patient data. Therefore, the effects of some study characteristics on the outcome could not be observed, such as sex of the patients, comorbidities, and cardiovascular therapies. Furthermore, although subgroup analysis suggested similar results in studies with multivariate analysis, we could not exclude the possibility that there are residual unadjusted factors that may confound the association between HGS and mortality in CAD patients. For instance, a diminished HGS has been correlated with inadequate nutritional status and heightened inflammatory response, both of which are linked to elevated mortality rates in patients diagnosed with CAD [40, 41]. Thus, the relationship between weakened HGS and heightened mortality risk in CAD patients may be confounded by malnutrition and systemic inflammation. Finally, as a meta‐analysis of observational studies, we could not establish a causative relationship between reduced HGS and increased risk of mortality in this patient population. Clinical trials are needed to determine if improving HGS could reduce the mortality risk in patients with CAD.

## 5 Conclusions

In conclusion, results of the meta‐analysis indicate that weak HGS at baseline may be associated with an increased mortality risk in patients with CAD, particularly in older patients. Although these results should be validated in large‐scale prospective studies, in view of the convenience, noninvasive nature, and repeatability of the methodology, measurement of HGS should be incorporated into the evaluation and management of patients with CAD. It would also be interesting to evaluate the influence of improving HGS on mortality risk in patients with CAD.

## Author Contributions

Meiling Xiao and Zhonghai Zhao designed the study. Meiling Xiao and Yu Lu performed the literature search, study selection, quality evaluation, and data collection. Meiling Xiao, Hongqiu Li, and Zhonghai Zhao performed statistical analyses and interpreted the data. Meiling Xiao drafted the manuscript. All authors revised the manuscript and approved the submission.

## Ethics Statement

The authors have nothing to report.

## Consent

The authors have nothing to report.

## Conflicts of Interest

The authors declare no conflicts of interest.

## Supporting information

Supporting information.

Supporting information.

Supporting information.

## Data Availability

The data sets generated and/or analyzed during the current study are available from the corresponding author on reasonable request.

## References

[1] C. W. Tsao , A. W. Aday , Z. I. Almarzooq , et al., “Heart Disease and Stroke Statistics‐2023 Update: A Report From the American Heart Association,” Circulation 147, no. 8 (2023): e93–e621. 36695182 10.1161/CIR.0000000000001123 PMC12135016

[2] P. Libby , G. Pasterkamp , F. Crea , and I. K. Jang , “Reassessing the Mechanisms of Acute Coronary Syndromes,” Circulation Research 124, no. 1 (2019): 150–160. 30605419 10.1161/CIRCRESAHA.118.311098 PMC6447371

[3] D. J. Medina‐Leyte , O. Zepeda‐García , M. Domínguez‐Pérez , A. González‐Garrido , T. Villarreal‐Molina , and L. Jacobo‐Albavera , “Endothelial Dysfunction, Inflammation and Coronary Artery Disease: Potential Biomarkers and Promising Therapeutical Approaches,” International Journal of Molecular Sciences 22, no. 8 (2021): 3850. 33917744 10.3390/ijms22083850 PMC8068178

[4] J. Atwood , “Management of Acute Coronary Syndrome,” Emergency Medicine Clinics of North America 40, no. 4 (2022): 693–706. 36396216 10.1016/j.emc.2022.06.008

[5] K. A. A. Fox , M. Metra , J. Morais , and D. Atar , “The Myth of ‘Stable’ Coronary Artery Disease,” Nature Reviews Cardiology 17, no. 1 (2020): 9–21. 31358978 10.1038/s41569-019-0233-y

[6] U. Ralapanawa and R. Sivakanesan , “Epidemiology and the Magnitude of Coronary Artery Disease and Acute Coronary Syndrome: A Narrative Review,” Journal of Epidemiology and Global Health 11, no. 2 (2021): 169–177. 33605111 10.2991/jegh.k.201217.001 PMC8242111

[7] M. Y. Henein , S. Vancheri , G. Longo , and F. Vancheri , “The Role of Inflammation in Cardiovascular Disease,” International Journal of Molecular Sciences 23, no. 21 (2022): 12906. 36361701 10.3390/ijms232112906 PMC9658900

[8] L. Pan , W. Xie , X. Fu , et al., “Inflammation and Sarcopenia: A Focus on Circulating Inflammatory Cytokines,” Experimental Gerontology 154 (2021): 111544. 34478826 10.1016/j.exger.2021.111544

[9] R. W. Bohannon , “Muscle Strength: Clinical and Prognostic Value of Hand‐Grip Dynamometry,” Current Opinion in Clinical Nutrition and Metabolic Care 18, no. 5 (2015): 465–470. 26147527 10.1097/MCO.0000000000000202

[10] Y. C. Wang , R. W. Bohannon , X. Li , B. Sindhu , and J. Kapellusch , “Hand‐Grip Strength: Normative Reference Values and Equations for Individuals 18 to 85 Years of Age Residing in the United States,” Journal of Orthopaedic & Sports Physical Therapy 48, no. 9 (2018): 685–693. 29792107 10.2519/jospt.2018.7851

[11] C. A. Celis‐Morales , P. Welsh , D. M. Lyall , et al., “Associations of Grip Strength With Cardiovascular, Respiratory, and Cancer Outcomes and All Cause Mortality: Prospective Cohort Study of Half a Million UK Biobank Participants,” BMJ 361 (2018): k1651. 29739772 10.1136/bmj.k1651 PMC5939721

[12] S. K. Jang , J. H. Kim , and Y. Lee , “Effect of Relative Handgrip Strength on Cardiovascular Disease Among Korean Adults Aged 45 Years and Older: Results From the Korean Longitudinal Study of Aging (2006‐2016),” Archives of Gerontology and Geriatrics 86 (2020): 103937. 31574451 10.1016/j.archger.2019.103937

[13] C. Zhuo , J. Zhao , Q. Wang , et al., “Assessment of Causal Associations Between Handgrip Strength and Cardiovascular Diseases: A Two Sample Mendelian Randomization Study,” Frontiers in Cardiovascular Medicine 9 (2022): 930077. 35990959 10.3389/fcvm.2022.930077 PMC9386423

[14] P. Lopez‐Jaramillo , J. P. Lopez‐Lopez , M. C. Tole , and D. D. Cohen , “Muscular Strength in Risk Factors for Cardiovascular Disease and Mortality: A Narrative Review,” The Anatolian Journal of Cardiology 26, no. 8 (2022): 598–607. 35924286 10.5152/AnatolJCardiol.2022.1586 PMC9403882

[15] J. W. Cook , L. M. Pierson , W. G. Herbert , et al., “The Influence of Patient Strength, Aerobic Capacity and Body Composition Upon Outcomes After Coronary Artery Bypass Grafting,” The Thoracic and Cardiovascular Surgeon 49, no. 2 (2001): 89–93. 11339458 10.1055/s-2001-11703

[16] J. Sanchis , C. Bonanad , V. Ruiz , et al., “Frailty and Other Geriatric Conditions for Risk Stratification of Older Patients With Acute Coronary Syndrome,” American Heart Journal 168, no. 5 (2014): 784–791.e2. 25440808 10.1016/j.ahj.2014.07.022

[17] J. A. Dodson , A. M. Hajduk , M. Geda , et al., “Predicting 6‐Month Mortality for Older Adults Hospitalized With Acute Myocardial Infarction: A Cohort Study,” Annals of Internal Medicine 172, no. 1 (2020): 12–21. 31816630 10.7326/M19-0974 PMC7695040

[18] B. Larcher , D. Zanolin‐Purin , A. Vonbank , et al., “Usefulness of Handgrip Strength to Predict Mortality in Patients With Coronary Artery Disease,” The American Journal of Cardiology 129 (2020): 5–9. 32580913 10.1016/j.amjcard.2020.05.006

[19] A. Tobe , A. Tanaka , Y. Shirai , et al., “Impact of Handgrip Strength on Clinical Outcomes After Percutaneous Coronary Intervention,” Journal of Atherosclerosis and Thrombosis 30, no. 9 (2023): 1115–1122. 36372431 10.5551/jat.63854 PMC10499459

[20] Z. Wang and H. W. Qin , “The Predictive Value of Grip Strength and Nutritional Status for Cardiovascular Adverse Events in Elderly Patients With Coronary Heart Disease,” Anhui Medical Journal 44, no. 4 (2023): 430–434.

[21] P. Kaul , K. P. Alexander , E. M. Ohman , et al., “Sex and Prognostic Significance of Self‐Reported Frailty in Non‐ST‐Segment Elevation Acute Coronary Syndromes: Insights From the TRILOGY ACS Trial,” Canadian Journal of Cardiology 35, no. 4 (2019): 430–437. 30935633 10.1016/j.cjca.2018.12.035

[22] M. L. Xu , F. F. Jiang , and H. Y. Liu , “Effect of Frailty on Short‐Term Prognosis of Hospitalized Elderly Patients With Coronary Heart Disease,” Contemporary Medicine 2022, no. 18 (2022): 68–71.

[23] D. F. Stroup , J. A. Berlin , and S. C. Morton , et al., “Meta‐Analysis of Observational Studies in Epidemiology: A Proposal for Reporting. Meta‐Analysis of Observational Studies in Epidemiology (MOOSE) Group,” JAMA 283, no. 15 (2000): 2008–2012. 10789670 10.1001/jama.283.15.2008

[24] G. A. Wells , B. Shea , D. O'Connell , et al., “The Newcastle‐Ottawa Scale (NOS) for Assessing the Quality of Nonrandomised Studies in Meta‐Analyses,” 2010, http://www.ohri.ca/programs/clinical_epidemiology/oxford.asp.

[25] J. Zhang and K. F. Yu , “What's the Relative Risk? A Method of Correcting the Odds Ratio in Cohort Studies of Common Outcomes,” JAMA 280, no. 19 (1998): 1690–1691. 9832001 10.1001/jama.280.19.1690

[26] J. Higgins and S. Green , *Cochrane Handbook for Systematic Reviews of Interventions Version 5.1.0* (London, UK: The Cochrane Collaboration, 2011), https://www.cochranehandbook.org/.

[27] J. P. T. Higgins and S. G. Thompson , “Quantifying Heterogeneity in a Meta‐Analysis,” Statistics in Medicine 21, no. 11 (2002): 1539–1558. 12111919 10.1002/sim.1186

[28] J. Higgins , J. Thomas , J. Chandler , et al., *Cochrane Handbook for Systematic Reviews of Interventions Version 6.2* (London, UK: The Cochrane Collaboration, 2021), https://training.cochrane.org/handbook.

[29] M. Egger , G. D. Smith , M. Schneider , and C. Minder , “Bias in Meta‐Analysis Detected by a Simple, Graphical Test,” BMJ 315, no. 7109 (1997): 629–634. 9310563 10.1136/bmj.315.7109.629 PMC2127453

[30] B. Ayça , D. Kafadar , M. Avsar , et al., “Lower Muscle Strength and Increased Visceral Fat Associated With No‐Reflow and High Gensini Score in STEMI,” Clinical and Applied Thrombosis/Hemostasis 23, no. 4 (2017): 367–373. 26494853 10.1177/1076029615613159

[31] Q. Xue , J. Wu , Y. Ren , J. Hu , K. Yang , and J. Cao , “Sarcopenia Predicts Adverse Outcomes in an Elderly Population With Coronary Artery Disease: A Systematic Review and Meta‐Analysis,” BMC Geriatrics 21, no. 1 (2021): 493. 34521369 10.1186/s12877-021-02438-w PMC8439080

[32] G. Tse , M. Gong , J. Nunez , et al., “Frailty and Mortality Outcomes After Percutaneous Coronary Intervention: A Systematic Review and Meta‐Analysis,” Journal of the American Medical Directors Association 18, no. 12 (2017): 1097.e1–1097.e10. 10.1016/j.jamda.2017.09.002 29079033

[33] S. B. Khoo , Y. L. Lin , G. J. Ho , M. C. Lee , and B. G. Hsu , “Association of Endothelial Dysfunction With Sarcopenia and Muscle Function in a Relatively Young Cohort of Kidney Transplant Recipients,” PeerJ 9 (2021): e12521. 34900434 10.7717/peerj.12521 PMC8614188

[34] C. S. L. Tuttle , L. A. N. Thang , and A. B. Maier , “Markers of Inflammation and Their Association With Muscle Strength and Mass: A Systematic Review and Meta‐Analysis,” Ageing Research Reviews 64 (2020): 101185. 32992047 10.1016/j.arr.2020.101185

[35] X. Zuo , X. Li , K. Tang , et al., “Sarcopenia and Cardiovascular Diseases: A Systematic Review and Meta‐Analysis,” Journal of Cachexia, Sarcopenia and Muscle 14, no. 3 (2023): 1183–1198. 37002802 10.1002/jcsm.13221 PMC10235887

[36] C. H. Chen , Y. J. Chen , H. P. Tu , M. H. Huang , J. H. Jhong , and K. L. Lin , “Benefits of Exercise Training and the Correlation Between Aerobic Capacity and Functional Outcomes and Quality of Life in Elderly Patients With Coronary Artery Disease,” The Kaohsiung Journal of Medical Sciences 30, no. 10 (2014): 521–530. 25438684 10.1016/j.kjms.2014.08.004 PMC11916713

[37] G. N. Marzuca‐Nassr , P. Seron , C. Román , et al., “A Hybrid Exercise‐Based Cardiac Rehabilitation Program Is an Effective Strategy to Improve Muscle Strength and Functional Exercise Capacity in Adults and Older People With Coronary Artery Disease,” Frontiers in Physiology 13 (2022): 948273. 35991183 10.3389/fphys.2022.948273 PMC9389047

[38] E. Coeckelberghs , R. Buys , K. Goetschalckx , V. A. Cornelissen , and L. Vanhees , “Prognostic Value of the Oxygen Uptake Efficiency Slope and Other Exercise Variables in Patients With Coronary Artery Disease,” European Journal of Preventive Cardiology 23, no. 3 (2016): 237–244. 25633586 10.1177/2047487315569410

[39] F. Cacciatore , P. Abete , F. Mazzella , et al., “Six‐Minute Walking Test But Not Ejection Fraction Predicts Mortality in Elderly Patients Undergoing Cardiac Rehabilitation Following Coronary Artery Bypass Grafting,” European Journal of Preventive Cardiology 19, no. 6 (2012): 1401–1409. 21933832 10.1177/1741826711422991

[40] A. S. Antonopoulos , A. Angelopoulos , P. Papanikolaou , et al., “Biomarkers of Vascular Inflammation for Cardiovascular Risk Prognostication,” JACC: Cardiovascular Imaging 15, no. 3 (2022): 460–471. 34801448 10.1016/j.jcmg.2021.09.014

[41] G. Arero , A. G. Arero , S. H. Mohammed , and A. Vasheghani‐Farahani , “Prognostic Potential of the Controlling Nutritional Status (CONUT) Score in Predicting All‐Cause Mortality and Major Adverse Cardiovascular Events in Patients With Coronary Artery Disease: A Meta‐Analysis,” Frontiers in Nutrition 9 (2022): 850641. 35614981 10.3389/fnut.2022.850641 PMC9125241

