# Supplementary material for: Association Between Handgrip Strength and Mortality of Patients With Coronary Artery Disease: A Meta‐Analysis
Source: Clin Cardiol. 2024 Jul 25;47(7):e24322. doi: 10.1002/clc.24322 (PMC11270052; doi:10.1002/clc.24322)

**Supplemental Figure 2** Funnel plots for the publication bias underlying the meta-analysis regarding the association between weak HGS and mortality risk of patients with CAD;


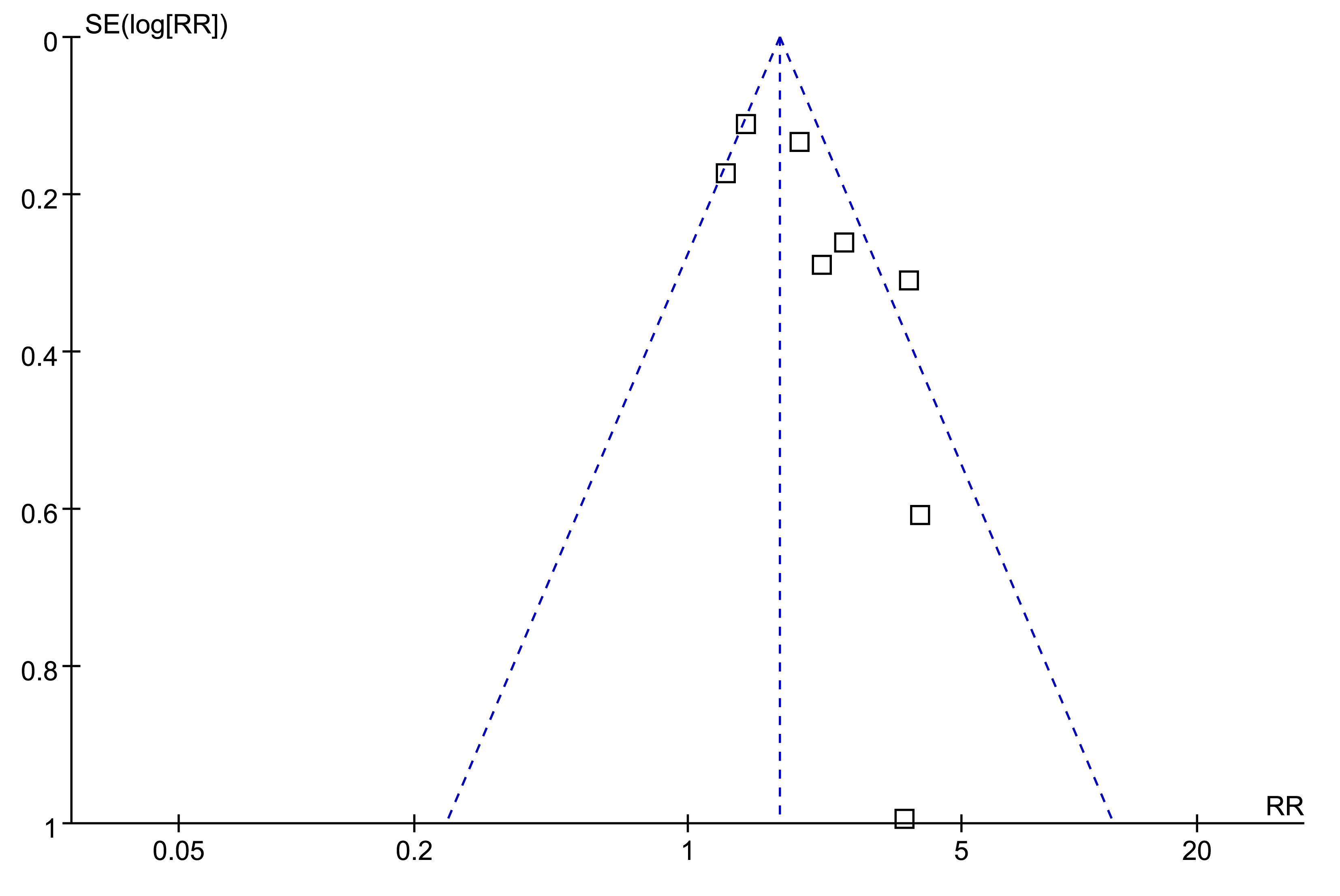

Supplement: Supplementary file 2 — Supporting information. [file CLC-47-e24322-s002.docx]
