# Supplementary material for: Association Between Handgrip Strength and Mortality of Patients With Coronary Artery Disease: A Meta‐Analysis
Source: Clin Cardiol. 2024 Jul 25;47(7):e24322. doi: 10.1002/clc.24322 (PMC11270052; doi:10.1002/clc.24322)
Supplement: Supplementary file 3 — Supporting information. [file CLC-47-e24322-s001.docx]

Supplemental Table 1 Study quality evaluation via the Newcastle-Ottawa Scale

| Study | Representativeness of the exposed cohort | Selection of the non-exposed cohort | Ascertainment of exposure | Outcome not present at baseline | Control for age and sex | Control for other confounding factors | Assessment of outcome | Enough long follow-up duration | Adequacy of follow-up of cohorts | Total |
| --- | --- | --- | --- | --- | --- | --- | --- | --- | --- | --- |
| Cook 2001 | 1 | 1 | 1 | 1 | 0 | 0 | 1 | 0 | 1 | 6 |
| Sanchis 2014 | 1 | 1 | 1 | 1 | 1 | 1 | 1 | 1 | 1 | 9 |
| Kaul 2019 | 0 | 1 | 1 | 1 | 1 | 1 | 1 | 1 | 1 | 8 |
| Dodson 2020 | 1 | 1 | 1 | 1 | 0 | 0 | 1 | 0 | 1 | 6 |
| Larcher 2020 | 1 | 1 | 1 | 1 | 1 | 1 | 1 | 1 | 1 | 9 |
| Xu 2022 | 0 | 1 | 1 | 1 | 1 | 1 | 1 | 0 | 1 | 7 |
| Tobe 2023 | 1 | 1 | 1 | 1 | 1 | 1 | 1 | 1 | 1 | 9 |
| Wang 2023 | 0 | 1 | 1 | 1 | 1 | 1 | 1 | 1 | 1 | 8 |
